# Challenges of College Students’ Ideological and Political and Psychological Education in the Information Age

**DOI:** 10.3389/fpsyg.2021.707973

**Published:** 2021-08-18

**Authors:** Xiaoqing He, Xiangrong Dong, Li Liu, Yulin Zou

**Affiliations:** ^1^School of Marxism, Chengdu Normal University, Chengdu, China; ^2^The Third Clinical Medical College of China Three Gorges University, Gezhouba Central Hospital of Sinopharm, Yichang, China

**Keywords:** information age, college students’ education, ideological and political education, psychological education, challenge

## Abstract

The purpose of the study is to analyze the current situation of ideological and political education and psychological education of college students, and explore the challenges faced by these two kinds of education in the information age. First, different research methods, such as literature research, questionnaire survey, and interdisciplinary research, are used to investigate and study the current situation of ideological and political education and psychological education of college students. Second, the survey data are analyzed to reveal the challenges they have encountered. The results show that there are many problems in ideological and political education, which need to be improved and strengthened. The Internet has a strong attraction for college students, but the purpose of getting online of many college students is not clear. Although it plays an important role in improving teaching quality by helping college students establish correct attitudes toward their study, values, life, and society, ideological and political education lack strong pertinence and effectiveness due to the professionalism of the teachers. And the negative impact of network information on ideological and political education of college students is also obvious. About 11.4% of the respondents believe that network information affects their physical and mental health. About 8.4% of the respondents believe that harmful information on the network is easy to induce students to behave wrongly. About 41% believe that computer games distract them from learning, which indicates that the negative impact of network information on college students cannot be underestimated, and it is imperative to strengthen the ideological and political education of college students. The research provides a useful reference for the ideological and political and psychological education in the information environment and helps to solve the problem in the ideological and political education and psychological education of college students.

## Introduction

The advent of the information age provides people with diversified ways to obtain information and a wide variety of interactive communication channels and has an important impact on all aspects of the lives of people (Hinz et al., 2017; McMurry et al., 2017; Sampogna et al., 2017). The characteristics of information communication, such as integration, sharing, virtual reality, and openness, always affect and change the learning process and lifestyles of college students. This not only provides opportunities but also brings greater challenges for ideological, political, and psychological education in colleges and universities in the information age (Hazelkorn, 2018; Williamson, 2018). Therefore, having a deep understanding of the challenges that ideological, political, and psychological education face in colleges and universities is of vital importance for them to retain vitality and creativity. Colleges and universities should strive to adapt to the information environment and flexibly use information technologies (Egert et al., 2018; Jeon et al., 2018). Only in this way can they innovate the ways and methods of ideological, political, and psychological education, achieve the goal of higher education, and realize the modernization and scientific development of ideological, political, and psychological education in colleges and universities in the new era (Kumlien et al., 2017).

Qian et al. (2018) found that psychological education, as an independent discipline, had developed for nearly a century in western countries, and it evolved from a career guidance movement in the early 20th century; Chen (2019) argued that, with the increasingly prominent drawbacks of pragmatism and the social problems brought by the drawbacks, the United States realized the importance of psychological education, and combined it with ideological and political education in practice, which mainly manifests in psychological discipline construction, professional counseling (Rogoza et al., 2018), and the construction of health and mental hygiene and psychological counseling institutions to guide the students toward social planning, achieve career development (Wu and Song, 2019), and have good interpersonal relations (Wu et al., 2019).

After years of theoretical research and practice, traditional ideological and political education has made some breakthroughs. Although it started a little late, psychological education quickly attracted the attention of the majority of scholars because of its special position in education. However, in the research on the topic, most scholars only focus on the innovative methods and opportunities of psychological education and ideological and political education and ignore their huge challenges. The innovation of this study is that the ideological and political education and psychological education of college students are studied based on a comprehensive survey and the characteristics of the information age. The challenges faced by ideological and political education and psychological education are summarized according to the research results, which provide a reference to the cultivation of college students (Shen et al., 2019).

## A Survey on the Status of Ideological, Political, and Psychological Education of College Students

### Methods

(1) Literature research method: first, a large number of materials, such as books, periodicals, and newspapers on ideological, political, and psychological education in colleges and universities in the information age, are searched, consulted, and reviewed (Kozlova, 2017; Djavari, 2018; Kim and Lim, 2019); and then the connotation, characteristics, challenges, and constraints of ideological, political, and psychological education are analyzed to explore new ways to solve the problem, having a general grasp of the status, the progress, and the focus of the topic. This can provide an academic reference and value guidance for the research.
In the information, age, ideological, political, and psychological, education in colleges and universities is full of various challenges. In the, *Innovative Development of Ideological and Political Education in Colleges and Universities in the Era of Big Data*, Wang Xuejian put forward the opportunities and challenges brought by the era of big data to ideological and political education in colleges and universities and discussed the countermeasures from a macro perspective. Li Chaojing, Xi Shexin, and Ye Jing pointed out in their article, *on the “trinity” mode of ideological and political education for postgraduates under the background of all media* – *taking Southeast University as an example*, that there are three major challenges faced by ideological and political education in colleges and universities in the information age, which include the dominant position, the educational methods, and the contents.
(2) Questionnaire survey method: the questionnaire survey is helpful in understanding the learning status of college students in the information age and mastering the influence of the information age on the ideological, political, and psychological education in colleges and universities. Besides, it is combined with the field survey method to exchange with scholars, professors, ideological and political teachers, psychological education authors, and student representatives in different fields (Nicholas et al., 2020; Deng et al., 2021), and summarize the opinions of different levels of people on the ideological, political, and psychological education in colleges and universities in the information age. After the literature is reviewed, and the status and the influences on ideological and political education and psychological education are analyzed, some scientific and reasonable methods and paths are proposed in response to the challenges.
(3) Interdisciplinary research method: the comparative analysis of the research results in the fields of communication, journalism, and information management is done, especially the analysis and exploration of the impact of information technology on the ideological, political, and psychological education in colleges and universities. And the development and changes of the discipline of ideological, political, and psychological education are integrated (Andersson Schwarz, 2017; Grates et al., 2019) to realize the interdisciplinary penetration and intersection, carry out research on interdisciplinary theories and achievements, and strive to provide theoretical and practical support for the existing constraints and challenges (You, 2017).

### Survey Design

The students majoring in ideological and political science and psychological education are selected from five universities as the survey subjects to explore the status and challenges of ideological and political education and psychological education of college students under information technology in China. These universities are social science organizations with strong comprehensive strength in the city and have a good teaching reputation in ideological, political, and psychological education. They are rich in educational resources and have a beautiful campus environment, professional teachers, and excellent students. The academic exchanges and scientific and technological communities develop harmoniously and are organized and formed among the universities, and the students in the universities have close contact with each other. Therefore, the comprehensive quality of students in the universities is almost at the same level. Stratified and random sampling methods are used in the survey to ensure the rationality of the data (McConnachie et al., 2018; Lin et al., 2019). Stratified sampling is mainly used to select a certain number of samples in different grades of different universities, while random sampling is used to pick up survey samples of different genders, ages, and majors. Besides, the field research is used to survey 65 students majoring in ideological and political science and 47 students majoring in psychological education to verify the rationality of the results. In addition, Cronbach’s α is used to test the reliability of the questionnaire. It overcomes the shortcomings of the partial split-half method and is the most commonly used reliability analysis method in the social science research field. If the answer to the question in the questionnaire is not comprehensive and does not conform to the convention, it will be invalid (Zhao et al., 2019; Liu and Chen, 2021).

**FIGURE 1:**
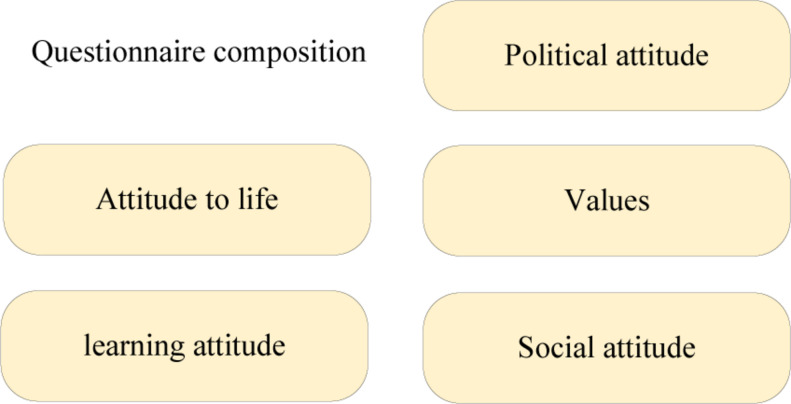
Questionnaire composition.

Since the research involves the ideological, political, and psychological education of college students, the questionnaire designed also includes two aspects. First, the content of ideological and political research (Lin and Hongtao, 2017) is designed from five aspects, namely the attitude toward politics, the view on values, the attitude toward life, the attitude toward society, and the attitude toward study. As college students in the information society, they should also learn and maintain the correct values, outlook on life, and attitude toward society and study. Second, the content of psychological education includes (Admiraal et al., 2017; Hidalgo-Mazzei et al., 2018; Dondé et al., 2019) student attention to the course, student engagement, sense of accomplishment of students, ability of students to use the knowledge, and the help to the students. In the process of investigation, attention of students to psychological education is investigated first, and then student engagement and the results after the participation are surveyed. Finally, the data of the questionnaire survey are carefully and rigorously sorted out. Figure 1 shows the questionnaire composition.

### Data Processing

**FIGURE 2:**
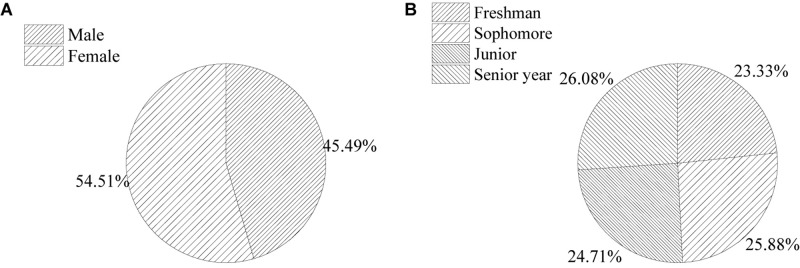
Basic information of the respondents [**(A)** the ratio of gender; **(B)** the ratio of grade].

The sample structure and the basic information of the respondents are shown in Figure 2.

The survey lasted 3 months from October to November of 2019, and the questionnaire was pre-investigated, returned, and improved. In December of the same year, large-scale analysis and research were conducted. About 550 questionnaires were distributed and 520 were recovered, with a recovery rate of 94.54%. However, 10 were invalid and incomplete. The number of effective questionnaires was 510, accounting for 92.72%. To verify the reliability, stability, and index system of the questionnaire data (Hidalgo-Mazzei et al., 2018), the quality of the questionnaire was tested (Klernäs et al., 2018; Fenwick et al., 2020; Petrie and Lynam, 2020), and SPSS 24.0 software was used to evaluate the α – reliability coefficient. The average value of Cronbach α was 0.871, which indicates that the questionnaire designed had strong internal consistency and stability with high credibility. The questionnaire is reasonable and effective and can be used as a tool for the research (Al-Ghamdi et al., 2018).

## Results of the Survey on the Ideological, Political, and Psychological Education of College Students

### The Investigation of Ideological and Political Education of College Students

In the information age, the use of the Internet has a subtle impact on the ideological and political concepts of college students. Therefore, the Internet habits of college students should be known before the status of ideological and political education in the information age is investigated.

**FIGURE 3:**
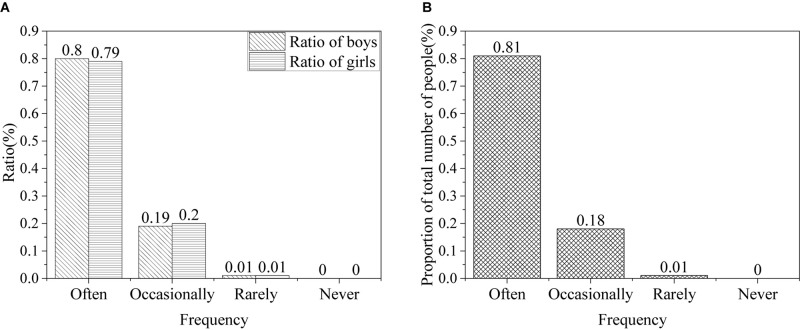
Frequency of college students using the Internet [**(A)** the ratio of males and females; **(B)** the ratio of all].

The frequency of Internet use by the college students is shown in Figure 3.

The results show that, in the information age, the Internet has an important and profound influence on the ideological and moral accomplishment of college students, and many begin to be addicted to the Internet. Among the students, about 80% of the students often use the Internet, 20% occasionally use, only 1% rarely use, and no one does not use it. Based on the results of the survey, college students have strong dependence on the Internet.

**FIGURE 4:**
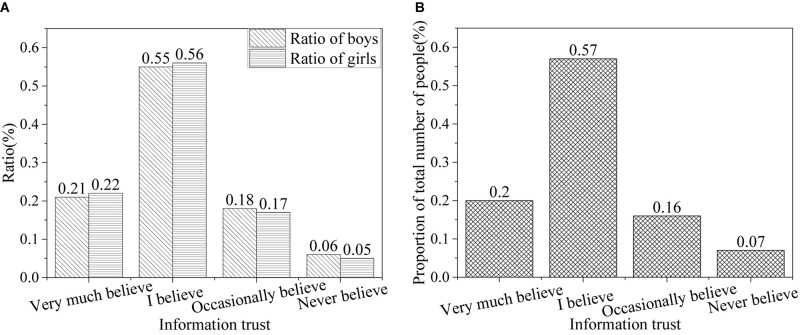
Trust of college students in the information on the Internet [**(A)** the ratio of males and females; **(B)** the ratio of all].

Figure 4 shows the results of trust of college student in the information on the Internet.

The survey results show that college students trust and have high dependence on the Internet, but they do not know about the risks and harm of it. About 20% of college students believe in network information, 56% of college students believe that network information is true, 17% of college students occasionally believe, and less than 1% never believe in network information. The continuous growth of network information brings a lot of convenience to users but also brings retrieval problems so that college students need more time to search and obtain the information needed. This is because the information on the internet has the characteristics of virtual reality, interactivity, convenience, and globality, which makes the learning and lives of college students inseparable from information technology. They are more inclined to use the network when looking for information. In addition, college students have the basic knowledge and skills to obtain network information, and also have the conditions to access the Internet, which makes college students dependent on the Internet. However, in the “Purpose of Access to Network Information” survey, 45% of students chose the option of “Entertainment,” 29.3% chose “communication,” and only 11% chose “Learning.” This proves that college students still have difficulties in getting information online and have no clear purpose of getting online, so it is urgent to strengthen the ideological and political education of college students.

**FIGURE 5:**
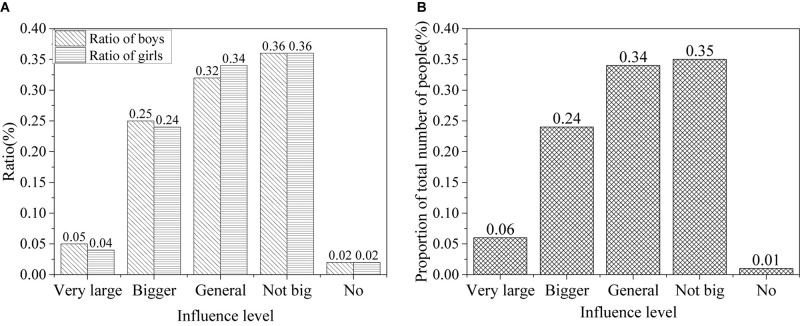
Influence of network information on the attitudes of college students toward politics [**(A)** the ratio of males and females; **(B)** the ratio of all].

In the information age, the influence of network information on political attitudes of college students is shown in Figure 5.

The survey shows that the Internet provides a good platform for college students to obtain information. About 5% of college students believe that network information has a great impact on their political attitudes, 25% believe that it has some impact, 33% believe that it has a certain impact, 35% believe that it has little impact, and only 2% believe that it has no impact. About 83.6% of the students surveyed believed that the network helps them obtain new information, and 77.2% relied on the network and believed that the network helps them know about the situation in China and foreign countries. College students can quickly obtain a large number of new information through the network, which greatly improves the efficiency of information. However, the results also show that some college students cannot make correct judgments on network information. About 25.9% believe that the network information is chaotic and difficult to distinguish between good and bad, and 13.2% believe that the bad information spread by the network can easily to harm their ideology and behavior. The survey shows that network information is mixed, and it should be selected when it is used.

**FIGURE 6:**
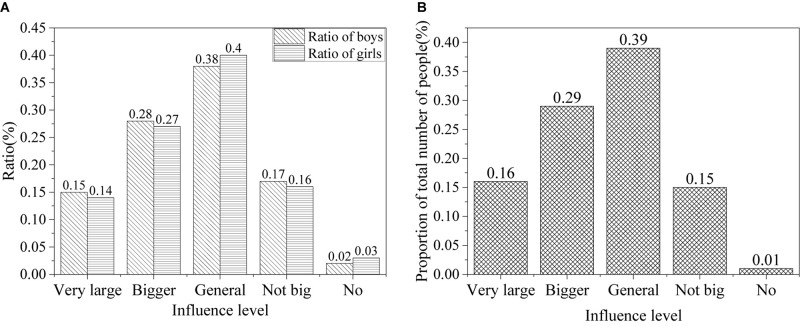
Impact of network information on values of college students [**(A)** the ratio of males and females; **(B)** the ratio of all].

The results of the influence of network information on values of college students in the information age are shown in Figure 6.

According to the results, the impact of the Internet on the values of college students is significant. About 15% of the college students think that the network information has a powerful influence on their values, about 28% argue that it has considerable impact on them, about 39% think it colors them, about 16% believe it has only a trivial effect, and only 2% think it means nothing to them. Overall, the Internet has a profound impact on values of college students in the information age.

**FIGURE 7:**
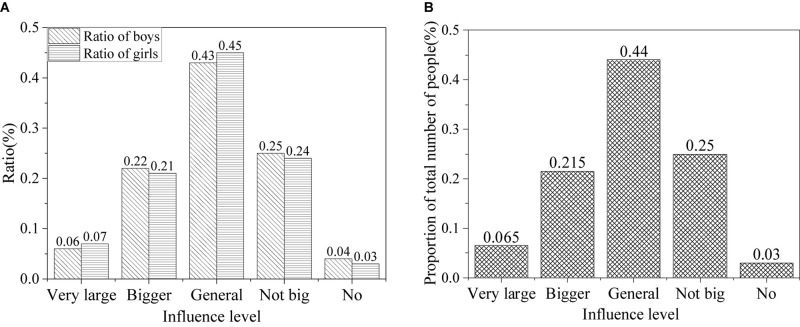
Influence of network information on attitude of college students toward life [**(A)** the ratio of male and female influence; **(B)** the ratio of all].

The results of the influence of network information on outlooks on life of college students in the information age are shown in Figure 7.

The above figure shows that the Internet has a significant impact on attitudes of college students toward life in the information age. About 6.5% of college students think that network information makes a big difference to their attitude toward life, about 21% think it has a certain impact, about 44% think it has an impact, about 24% think it has little impact, and only 3.5% think it has no impact.

**FIGURE 8:**
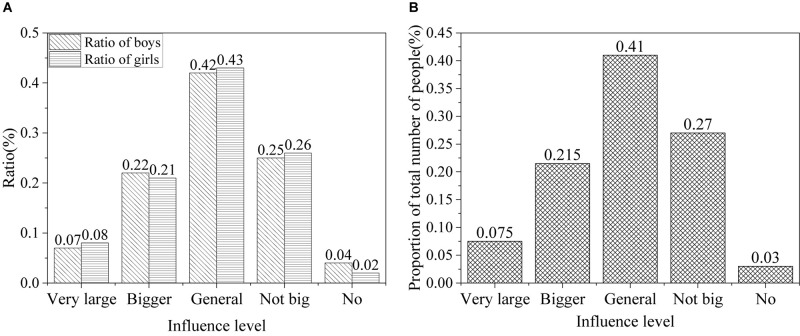
Influence of network information on attitude of college students toward society [**(A)** the ratio of males and females; **(B)** the ratio of all].

The results of the influence of network information on attitude of college students toward society in the information age are shown in Figure 8.

According to the research results, it is found that the Internet has an obvious impact on attitudes of college students toward society in the information age. About 7.5% believe it has a serious influence on them, 21% think it does affect them, 42% think it has a certain effect, 26% argue that it has little impact, and only 3% think it never affects their attitude toward society.

**FIGURE 9:**
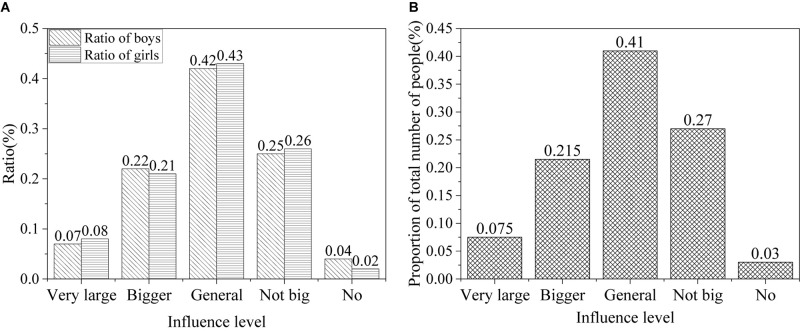
Influence of network information on the attitudes of college students toward study [**(A)** the ratio of male and female influence; **(B)** the ratio of all].

The results of the influence of network information on the attitudes of college students toward study in the information age are shown in Figure 9.

Learning is an important mission for college students. Similarly, the influence of the Internet on attitude of college students toward study is great in the information age. About 7.5% of college students think that network information has a major influence, 21.5% argue that it has some impact, 42% think that it has an impact, 26% think that it has little impact, and only 2.5% of the students think it affects nothing.

### Results of the Survey on Psychological Education of College Students

In the information age, the development of networks not only affects the ideological and political education of college students but also the psychological influence of students.

**FIGURE 10:**
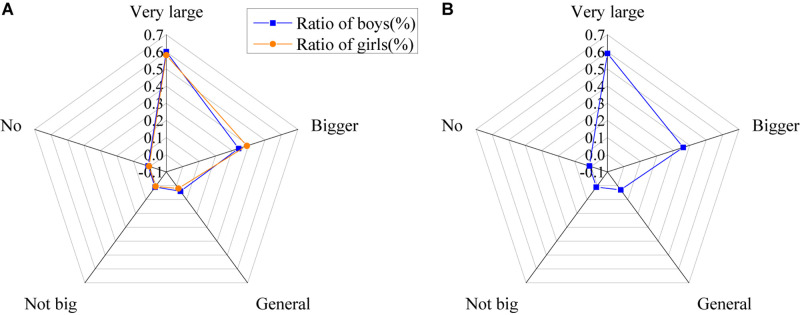
Influence of network information on the psychology of college students [**(A)** the ratio of males and females; **(B)** the ratio of all].

First, the results of the influence of network information on the psychology of college students are shown in Figure 10.

If college students contact internet information daily, the impact of the information on their psychology will inevitably occur. According to the results, 59% of college students think the impact of network information on them is significant, 37% say that its effect is big, and 3% argue that it has a general impact. And the number of the students who believe that it has little or no impact accounts for 1%, respectively.

**FIGURE 11:**
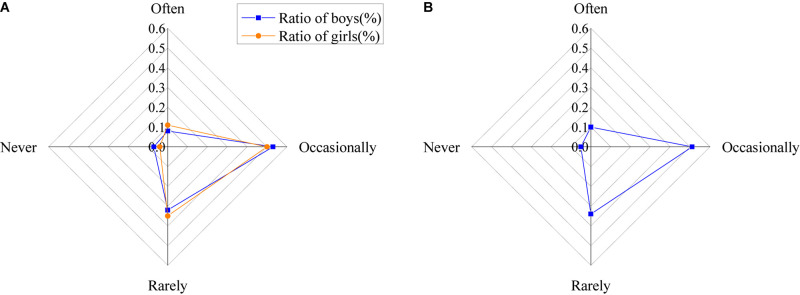
Initiative enthusiasm college students of studying psychological knowledge [**(A)** the ratio of males and females; **(B)** the ratio of all].

The results of initiative enthusiasm of college students of studying psychological knowledge are shown in Figure 11.

Although college students think that learning psychological knowledge is very important, they still lack certain enthusiasm for it consciously and actively. As shown in the figure, about 10% of the students often learn relevant knowledge, 52% of the students occasionally learn relevant knowledge, 34% of the students seldom learn relevant knowledge, and 6% the students never learn relevant knowledge consciously.

**FIGURE 12:**
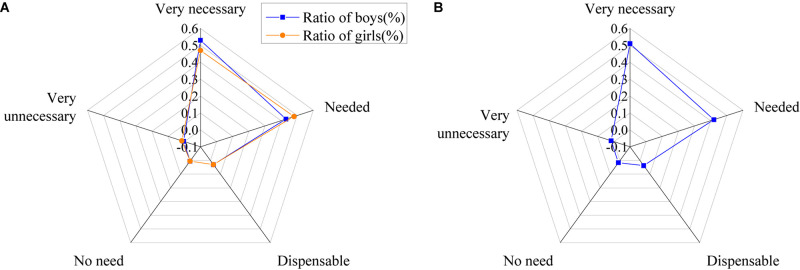
Necessity of learning psychological knowledge [**(A)** the ratio of men and women; **(B)** the ratio of all].

In the information age, the results of the necessity of learning psychological knowledge are shown in Figure 12.

The figure shows that about 52% of the students think it is important to learn psychological knowledge, 47% think it is necessary, 3% think it is an optional extra, and only about 1 and 1.5% think it is unnecessary and has no need to learn, respectively.

**FIGURE 13:**
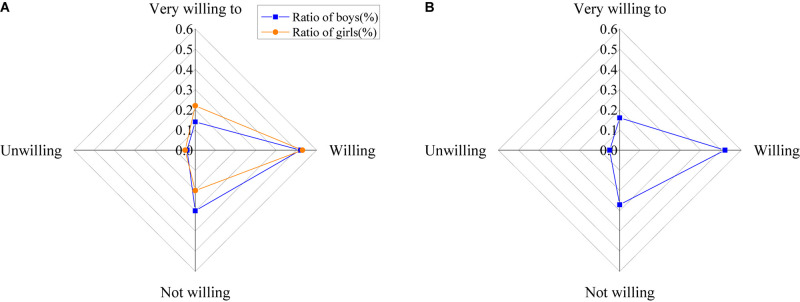
Willingness of college students to participate in psychological education [**(A)** the ratio of the males and females; **(B)** the ratio of all].

The willingness of the college students to participate in psychological education is shown in Figure 13.

The figure shows that 19% of the students are adequately willing to participate in activities related to psychological education, 52% are more likely to participate in the activities, 28% have little desire for them, and about 4.5% are not willing to participate.

**FIGURE 14:**
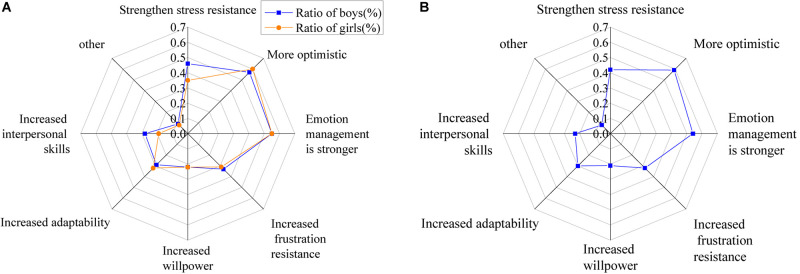
Benefits that the psychological course brings [**(A)** the ratio of males and females; **(B)** the ratio of all].

The benefits that the psychological course brings are shown in Figure 14.

The figure shows that if college students participate in psychological education activities, it will bring them something good. The course strengthens their stress resistance, makes them more optimistic, and enhances their emotional management. Besides, it also enhances their frustration resistance, improves their adaptation capacity and willpower, and promotes their interpersonal communication.

**FIGURE 15:**
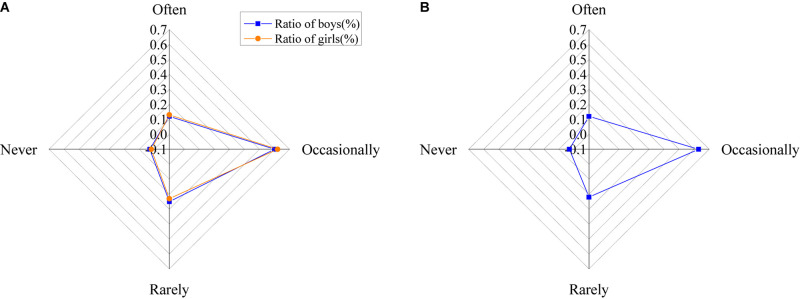
The use of psychological knowledge by college students [**(A)** the ratio of males and females; **(B)** the ratio of all].

The results of the use of college students of psychological knowledge are shown in Figure 15.

The figure shows that 12.5% of college students often use psychological knowledge, 62% occasionally use it, 24% seldom use it, and only 2.5% never use psychological knowledge in life.

**FIGURE 16:**
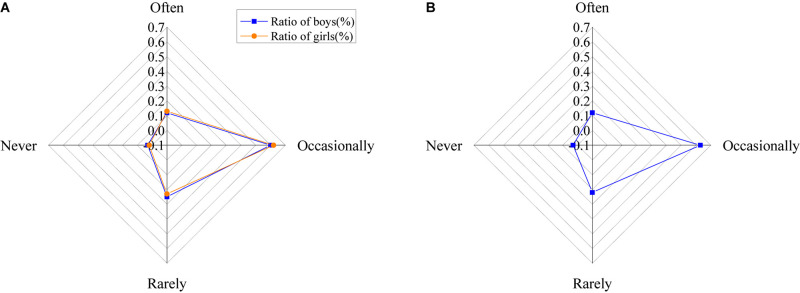
Help of psychological education to college students [**(A)** the ratio of males and females; **(B)** the ratio of all].

The help of psychological education to college students is shown in Figure 16.

The results of psychological education on college students show that about 14% of college students think that psychological education has great help to themselves, 48% think that it has some help, 35% of students think that it has a little help, and 3% think that it has no help.

The survey results show that the influence of the internet on college students is various, and the ideological and political education of college students has made some progress. However, there are still many problems in ideological and political education, which need to be improved and strengthened. The internet has a strong attraction for college students, but many college students do not have a clear purpose for getting online. The internet provides college students with a lot of information, but some college students have difficulties in picking up useful and accurate information. It has a positive and negative influence on the ideology, attitudes toward politics, and behavior of college students. Although it plays an important role in improving the teaching quality of the course and helping college students establish correct attitudes toward politics, values, life, society, and study, ideological and political education lacks strong pertinence and effectiveness because of the profession and high demand for the construction of academic staff.

### Challenges of Ideological, Political, and Psychological Education

The data of the surveys show that the ideological, political, and psychological education of college students in China is experiencing severe challenges under the information environment. First, ideological and political education plays an important role in cultivating talents, but the current teaching approach has a general purpose; second, ideological and political education should be in tune with the times, whereas the teachers are the bottleneck that slows the development of ideological and political education. In terms of psychological education, colleges with the primary conditions cannot meet the demand of the development of students; besides, the disharmonious relationship between virtual and reality that the information age brings about affects the psychological education of college students.

## Conclusion

A comprehensive investigation is carried out on the challenges that the ideological, political, and psychological education of college students encounter in the information age. First, the literature research method is used to make a preliminary study on the previous research on the ideological, political, and psychological education of college students. Second, the questionnaires were distributed and an interview was employed to study the students in five universities. Finally, the effectiveness of the data was analyzed. The results show that there are serious problems and challenges in the ideological, political, and psychological education in colleges and universities in the information age. The ideological and political education is not targeted and efficient, and psychological education has the problems of a mismatch between supply and demand, as well as virtual and reality. Although a thorough investigation of the current situation of ideological, political, and psychological education of college students was conducted in the context of informatization and its challenges were explored, there are still shortcomings in this study. These will be the content of the upcoming study. This study provides a reference to the research on the challenges faced by ideological, political, and psychological education of college students in the context of informatization.

## Data Availability Statement

The raw data supporting the conclusions of this article will be made available by the authors, without undue reservation.

## Ethics Statement

The studies involving human participants were reviewed and approved by the Ethics Committees of Chengdu Normal University and Gezhouba Central Hospital of Sinopharm. The patients/participants provided their written informed consent to participate in this study. Written informed consent was obtained from the individual(s) for the publication of any potentially identifiable images or data included in this article.

## Author Contributions

All authors listed have made a substantial, direct and intellectual contribution to the work, and approved it for publication.

## Conflict of Interest

The authors declare that the research was conducted in the absence of any commercial or financial relationships that could be construed as a potential conflict of interest.

## Publisher’s Note

All claims expressed in this article are solely those of the authors and do not necessarily represent those of their affiliated organizations, or those of the publisher, the editors and the reviewers. Any product that may be evaluated in this article, or claim that may be made by its manufacturer, is not guaranteed or endorsed by the publisher.
